# Illinois

**Published:** 1896-12

**Authors:** 


*Illinois.* Drs. Burrill and McIntosh, of Champaign, are investigating an outbreak of disease, of a very fatal character, among the hogs in Champaign County. Those that have died are said to have been inoculated with the Detmer hog-cholera cure.

